# Knowledge of and Adherence to the Cyanide Code Among Small-scale Gold Miners in Northern Tanzania

**DOI:** 10.5696/2156-9614-7.14.4

**Published:** 2017-06-22

**Authors:** Elias C. Nyanza, Petro Yohana, Deborah S.K. Thomas, Wilfreda E. Thurston, Eveline Konje, Deborah Dewey

**Affiliations:** 1 School of Public Health, Catholic University of Health and Allied Sciences, Mwanza, Tanzania; 2 Department of Community Health Sciences, Cumming School of Medicine, University of Calgary, Calgary, Canada; 3 Department of Geography & Environmental Sciences, University of Colorado Denver, Denver, CO, USA; 4 Department of Paediatrics and Owerko Centre at the Alberta Children's Hospital Research Institute, Cumming School of Medicine, University of Calgary, Calgary, Canada

**Keywords:** cyanide, code of practice, gold mining, occupational health, safety, ecosystem

## Abstract

**Background.:**

Tanzania has seen explosive development in small scale gold mining (SGM) operations. Recently, the use of cyanide has become more common in SGM, especially in the reprocessing of mercury-amalgamated tailings from artisanal mining sites.

**Objectives.:**

The primary objective of this study was to examine the level of knowledge and adherence to the Cyanide Code among workers and managers at SGM operations in northwestern Tanzania that use cyanide for gold extraction, focusing on workers' safety.

**Methods.:**

A cross-sectional study of workers and managers at 17 selected SGM sites was conducted. A random-cluster approach was used to recruit 215 mine workers and 23 mine managers who worked at the same sites for more than three months. Individuals participated in structured face-to-face interviews. Site evaluation checklists were also administered to assess adherence.

**Results.:**

The majority of the SGM workers (61.4%, n=132) were not aware of the Cyanide Code. Among the mine managers, 64.2% (n=15) were aware of the Cyanide Code. Fifty-four percent of workers and 39.1% of managers did not adhere to the Cyanide Code. Workers who reported being trained on the Cyanide Code were significantly more likely to have knowledge about the Cyanide Code guidelines compared to untrained workers (adjusted odds ratio =20.3, confidence interval: 7.5 – 54.8).

**Discussion.:**

Wide variations in knowledge of and adherence to the Cyanide Code were found. A manager's knowledge of Cyanide Code was significantly associated with workers' knowledge. High worker and manager knowledge was associated with increased site safety performance. Even though all the SGM sites were physically visited, some potentially hazardous practices may not have been revealed by managers and workers because of fear of possible regulatory actions due to disclosure of concerns related to their operation's safety compliance.

**Conclusions.:**

The limited knowledge of the Cyanide Code among workers and managers, combined with poor adherence to cyanide waste management practices, indicates that there is a need for education, health promotion and sensitization among workers and managers to improve worker safety and minimize environmental health impacts.

**Participant Consent:**

Obtained

**Ethics Approval:**

Ethical approval was obtained from the Conjoint Catholic University of Health and Allied Sciences and Bugando Medical Centre Research Review and Ethics Committee (Ref. BREC/001/35/2014). Permission to conduct research in Geita District was obtained from the respective authorities at the regional and district levels and from owners of the SGM sites.

## Introduction

Many low- and middle-income countries have seen explosive development of the artisanal and small-scale gold mining industry (ASGM). While no strict definition exists differentiating artisanal from small-scale mining, artisanal mining typically refers to informal processes that are predominantly manual and have low levels of production. Those that are more organized with some mechanization and higher production levels are referred to as small scale gold mining (SGM) operations.^1^,^2^ Small scale can also refer to the size of the operation; for example, Tanzania's 2010 Mining Act recognizes SGMs as mining operations whose capital investment is less than US$ 100,000.^3^ There are more than 16 million active gold miners participating in ASGM in more than 55 countries worldwide; and ASGM provides a direct and/or indirect source of livelihood for more than 100–180 million people.^1^,^2^,^4–6^ Tanzania has the second largest population directly involved in ASGM (more than 1.5 million individuals) in Africa. It provides approximately 15 million people with a livelihood and accounts for about 10% of the gold produced in the country.^5^,^7^ The number of people either directly or indirectly employed in this sector continues to increase due to an increase in gold prices and because of the limited number of other livelihood options in many rural areas of Tanzania.^5^,^8^

In Tanzania, ASGM activities are conducted haphazardly without regard for environmental, occupational or community exposures.^7–11^ Over the last two decades, much of the global concern related to ASGM has focused on the rudimentary use of mercury, and how exposure could be minimized through the use of cleaner technologies.^5^,^10^,^12^,^13^ Traditionally, ASGM gold miners have used mercury amalgamation, a simple and inexpensive, but inefficient way to extract gold, as this technique results in less than 40% of the gold being extracted from the ore. As a result, large quantities of gold are left in the tailings, as well as significant amounts of unrestricted mercury.^9^,^11^,^13^,^14^

In recent years, there has been an increase in gold mining investment in the SGM industry accompanied by a shift from the use of mercury to the use of cyanide technology for gold extraction. The use of cyanide for gold extraction is a relatively efficient process.^14^,^15^ While cyanide has been commonly used by large scale gold miners, this technology is now popular and is being used in the SGM industry worldwide.^8^,^14–16^ This is the case in northern Tanzania, including the Lake Victoria basin, where cyanide is used to leach the gold from the mercury amalgamated tailings created by artisanal and/or other small-scale gold miners.^5^,^15^,^16^

**Abbreviations**
*AOR*: Adjusted odds ratio
*ASGM*: Artisanal and small-scale gold mining industry
*CI*: Confidence interval
*SGM*: Small-scale gold mining

The leaching of gold from the mercury amalgamated tailings with cyanide could increase the health risks associated with mercury and cyanide exposure to miners and the surrounding communities.^14–18^ When mercury combines with cyanide it forms highly soluble complexes such as anionic mercury (II) cyanide ([Hg(CN)4]^2−^), which is stable at a pH above 8.5, or mercury (II) cyanide (Hg(CN)_2_), which is stable at a pH below 7.8.^19^ However, at a pH above 7.8, mercury becomes not only soluble but bioavailable and more easily methylated.^20^,^21^ This could result in mercury bioaccumulation in the environment and potential harm to human and animal populations who reside in surrounding communities.^14–17^,^19^,^23^

Cyanide and its compounds are potentially poisonous and can cause significant damage to people and the environment if not handled carefully.^14^,^17^ In many SGM operations, waste management is reported to be inadequate and workers handle cyanide and cyanide compounds without protective gear.^14^,^15^ For example, in Ecuador, dumping of cyanide waste has been found to cause serious damage to the environment and to human health.^23^ In order to minimize these risks, the management of cyanide is one of the key challenges faced by the gold mining industry worldwide.^5^,^14^

The mining industry has an obligation to prevent the release of toxic chemicals into the environment, ensuring that humans, birds, animals, and aquatic life are not endangered by the storage and discharge of wastewater.^15^,^24^ In response to incidents such as those in Ecuador, and to assist the gold mining industry in improving their management of cyanide, the Cyanide Code was developed.^25^ The Cyanide Code consists of nine principles; the first five provide guidance on cyanide management during production, transportation, handling and storage, operations, and decommissioning. The last four provide guidance on worker safety, emergency response training, and in conducting dialogue (i.e., engagment in public consultation and disclosure).

The Cyanide Code is managed by the International Cyanide Management Institute that has developed a risk-based management process that focuses on improving the management of cyanide application at both large and small-scale gold mining sites to assist in the protection of human health and the reduction of environmental impacts.^5^,^25^ Compliance with the Cyanide Code is entirely voluntary.^25^ The Cyanide Code is intended to complement an operation's existing regulatory requirements. Thus, to be in compliance with the Cyanide Code, the rules, regulations and laws of the applicable political authority must be followed.

In Tanzania, the Cyanide Code was formally introduced in 2006 and was immediately adopted by the large-scale gold mining industry. In later years, a few of the SGM operators who were using cyanide as a lixiviant – a liquid medium to selectively extract gold from the ore – also implemented the Cyanide Code.^8^,^18^,^26^ Currently, government mining officers are obliged to provide guidance to all registered SGM operations using cyanide on how to use and adhere to the Cyanide Code throughout the entire gold production process; however, there are many other SGM operations that are not registered with the government.^3^ Implementation and adherence to the Cyanide Code by the gold mining industry in Tanzania is required under the following mining and environmental management acts and regulations: 1) The Mining (Environmental Protection for Small Scale Mining) Regulations of 2010; 2) The Mining (Safety Occupational Health and Environmental Protection) Regulations of 2010; 3) Industrial and Consumer Chemical (Management and Control) Act of 2003; 4) The Environmental Management Act of 2004; 5) The Environmental Management (Hazardous Waste Control) Regulations of 2009; and 6) The Tanzania Occupational Health and Safety act of 2003.^3^,^27–29^ The Tanzanian government has also recognized the importance of the SGM industry by establishing and enacting several policies and regulations through the Ministry of Energy and Minerals. These include the Tanzanian Mining Policy of 1997, the Mining Act of 1998, and the Mining Act of 2010, which legalized the SGM industry, and established a set of basic environmental and safety standards and a new permitting system.^3^,^29^ Although these regulations are in place, compliance in the SGM industry in Tanzania is limited.^14^,^17^,^30^

The Public Health Act of Tanzania stipulates that waste producers are expected to take responsibility for the collection, transportation, storage, and treatment of waste.^27^ The Act delegates waste management to producers of waste by requiring them to follow existing waste management standards and procedures in handling waste produced by Tanzanian mines. However, compliance with health and safety codes and codes of practice, such as the Cyanide Code, remain undocumented.

Reports from SGM mining areas in Tanzania suggest a lack of enforcement of environmental safety regulations, as well as occupational health and safety regulations.^10^,^31^ Furthermore, many of the individuals involved in SGM lack education and training on mine site occupational health and safety and do not use protective measures.^5^ In Tanzania, training in cyanide management – knowledge about safe methods of handling, using and disposing of cyanide residual – in most cases remains the responsibility of SGM owners in collaboration with regulatory authorities, and may not be a priority among mining managers and owners; however, this has not been studied.^3^,^27–29^ Essential equipment for safe mining practices is frequently absent from SGM mining sites.^5^ Finally, efforts to protect surrounding communities are very limited or nonexistent.^10^ This is consistent with the findings of a recent study in northern Tanzania that suggested that a lack of knowledge among ASGM miners contributed to an absence of environmental monitoring and poor waste management practices among miners.^31^ The findings of this study highlighted the need for community health education and policy changes to safeguard the health of the miners and the communities located near gold mining sites.

Improving health and safety for workers and reducing the environmental risks associated with the use of cyanide in SGM operations depends upon the workers' and managers' knowledge of, motivation and skills in implementing the Cyanide Code.^14^ However, despite the increased use of cyanide in the SGM industry in Tanzania, the extent of workers' and managers' knowledge regarding the safety practices associated with cyanide use and their adherence to the Cyanide Code is unknown. The primary purpose of this study was to investigate knowledge of and adherence to the Cyanide Code among workers and managers involved in SGM operations in northwest Tanzania, with a focus on worker health and safety.

## Methods

### Setting

This study was conducted in Geita District, which has a population of 807,617.^32^ This district was chosen because it has the largest number of ASGM and SGM gold mining operations around the Lake Victoria goldfield and is one of Tanzania's main gold producing districts.^9^,^11^ It has four major small scale gold mining centres, Nyarugusu, Nyakagwe, Mgusu and Rwamagasa, and there are more than 21 SGM operations using cyanide to leach mercury amalgamated tailings. These operations provide direct employment to more than 560 workers.^33^ Note that this study focused on SGM, and did not include artisanal gold miners as the latter do not currently use cyanide in mining processes.^26^

### Design

This cross-sectional study conducted face-to-face interviews with SGM workers and managers between June 2014 to February 2015. A semi-structured questionnaire adapted from a similar questionnaire used in our previous research was used to examine workers' and managers' knowledge of the Cyanide Code.^31^ The questionnaire consisted of seven questions (*Supplemental Material 1*). The percent correct of the seven questions was calculated to provide a total score. Individuals with a total percent correct score of ≥ 50% were classified as knowledgeable and those with a percent correct score of < 50% were classified as not knowledgeable.

Knowledge on adherence to the COO among mining workers and managers was also examined using a semi-structured questionnaire administered in the form of a face-to-face interview (*Supplemental Material 2*). Mine workers and managers with a total percent correct of ≥ 50% were classified as knowledgeable about adherence to the Cyanide Code and those who scored < 50% were classified as not knowledgeable.

Adherence of SGM industry sites to the Cyanide Code was also assessed using two site audit checklists: 1) a 17-item checklist developed using the requirements by the International Cyanide Management Institute (*Supplemental Material 2 and 3*) an observation checklist adapted from Rosia Montana Gold Corporation (*Supplemental Material 4*).^25^,^34^ A SGM site with a score ≥ 50% was considered to be adhering to the Cyanide Code. If it obtained a score of <50%, it was classified as not adhering to the Cyanide Code. A pictorial presentation of the SGM site operation is presented in Supplemental Material 5.

The questionnaires and checklists were translated from English to Swahili (the primary language of most of Tanzania) and back translated to English using a second translator to ensure accuracy and language consistency. Various tools (viz., semi-structured questionnaires for managers and workers, and standardized checklists) were used to increase the rigor of the study and ensure the reliability of the findings.

The study tools were pre-tested at Ishokela mining sites (i.e., SGM operations that use cyanide) in Misungwi District to ensure that the interview questions and checklists had face validity and were understood by the interviewers and respondents. The data collected at Ishokela was not included in the current study. A total of 57 workers and 6 managers from four sites participated in these pre-test activities. Unclear and ambiguous questions were modified and/or rephrased where necessary. Two researchers on our team (PY and ECN) also completed the checklists independently to assess usability. The scores of two team members were then compared and discrepancies were resolved through discussion based on the evidence provided and changes were made to the checklists as necessary.

Twenty-one SGM sites in Geita District that used cyanidation technology and had a cyanide leaching permit were invited to participate in this study; 19 sites agreed to take part and 2 sites declined. A total of 215 workers and 23 managers who had worked at their respective sites for more than three months agreed to participate. None of the participants withdrew from the study. The criteria of working at a specific site for a minimum of three months was used to ensure that participants were familiar with the routine working practices at that SGM site.

A random sampling technique was used to recruit SGM workers from the list of all the workers at each of the SGM sites. Each individual was assigned a unique number. The numbers were then mixed up in a closed container to give each individual an equal chance of being selected. Since each of the SGM sites had either one or two managers, all managers were invited to participate in the study provided they had been in their position for more than three months.

### Analysis

Data were analyzed using the Statistical Package for the Social Sciences (SPSS) v. 17.0^™^. Chi-square tests or Fisher's exact tests were used as appropriate to investigate the associations between socio-demographic variables (for example, age, sex, marital status, education, work station, number of years at work, number of hours worked a day, and any training on cyanide management) and knowledge of the Cyanide Code. Sociodemographic information, training and years of work experience were used to calculate the odds of being knowledgeable. Results were considered statistically significant when p-values were < .05 and were reported with 95% confidence intervals.

### Ethical Approvals

Ethical approval was obtained from the Conjoint Catholic University of Health and Allied Sciences and Bugando Medical Centre Research Review and Ethics Committee (Ref. BREC/001/35/2014). Permission to conduct research in Geita District was obtained from the respective authorities at the regional and district levels and from owners of the SGM sites. Participation in the study was voluntary. Written consent was sought from participants prior to recruitment. Participation in this study was on a voluntary basis. No names of respondents have been used in this article or any other reports. During the fieldwork, numbers were assigned to participants to ensure anonymity.

## Results

### Socio Demographic Characteristics of Respondents

#### Mine Workers

Table 1 provides details on the socio-demographic and work characteristics of the workers. A total of 215 workers aged between 18–59 years participated in the study. Most of the respondents (n=93, 43.3%) were 20–30 years of age. More than 89% (n=192) were male and the majority of the respondents were married (n=163, 75.8%). Most of the respondents had been working in the cyanide (41.4%, n=89) and processing (39.5%, n=85) sections of the mining sites for more than 5 years. Almost all of the respondents (n=208, 96.3%) worked for more than 8 hours a day. More than half of the respondents (n=125, 58.1%) had completed primary school education.

**Table 1:**
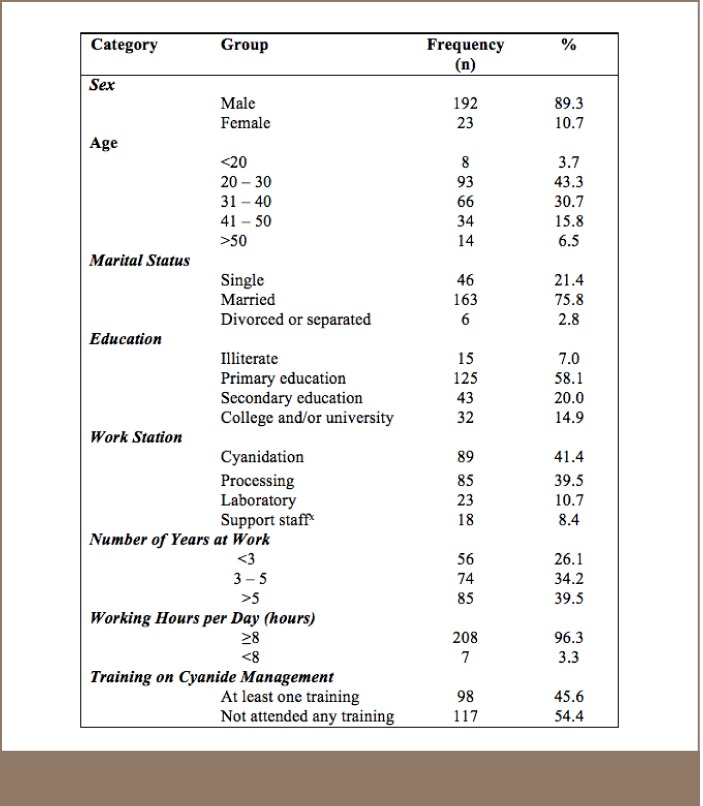
Socio Demographic and Work Characteristics of SGM Workers

#### Mine Managers

A total of 23 managers agreed to complete the survey. The 23 managers ranged in age from 31 and 57 years, and all were male. A majority of the managers (60.9%, n=14) had completed primary school and 39.1% (n=9) had completed secondary or tertiary education. More than half of the managers (52.2%, n=12) had more than 6 years of work experience in the SGM industry (*Table 2*).

**Table 2:**
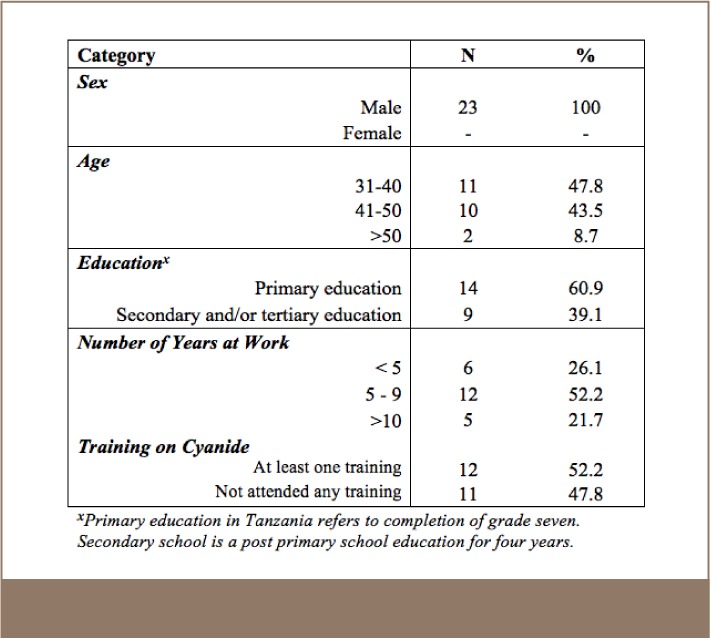
Socio Demographic and Work Characteristics of SGM Managers

### Knowledge about the Cyanide Code among Workers and Managers

A majority of the SGM workers (61.4%, n=132) were not aware of the Cyanide Code. Among the mine managers interviewed, 64.2% (n=15) were aware of the Cyanide Code. Age, marital status, education level, training at the mining site, and work experience of mining workers were found to significantly increase the odds of being knowledgeable of the Cyanide Code (*Table 3*). Individuals (i.e., workers and managers) with post-secondary education had more knowledge of the Cyanide Code compared to individuals in the same occupations with lower levels of education (adjusted odds ratio (AOR) = 11.2; confidence interval (CI) = 4.4 – 13.6). Training at the worksite, however, had the greatest association with knowledge of the Cyanide Code (AOR = 20.3; CI = 7.5 – 54.8). Manager knowledge about the Cyanide Code was associated with worker knowledge (χ^2^ = 4.8, p = 0.02).

**Table 3:**
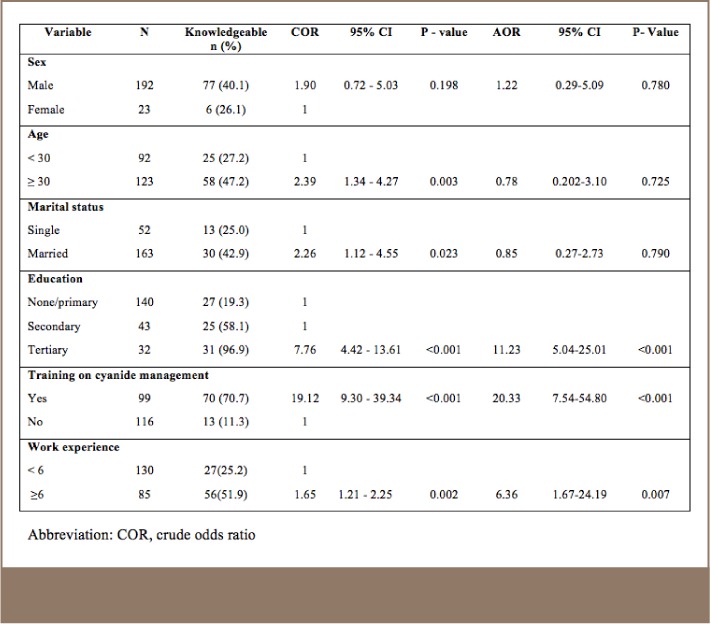
Mine Worker Knowledge of the Cyanide Code

#### Knowledge About Adherence to the Cyanide Code Among Workers and Managers

Of the mine workers, 46% (n = 99) were classified as being knowledgeable about adherence measures in the Cyanide Code and 60.9% (n=14) of the mine managers were classified as knowledgeable. Because of the small number of managers, correlates were not investigated. Among the mine workers, male workers were found to have more knowledge about adherence to the Cyanide Code compared to female workers (AOR = 3.1; CI=1.2 – 7.9). Number of years of work experience was not significantly associated with knowledge about adherence to the Cyanide Code among the mine workers (AOR = 0.99; CI=0.48 - 2.09) (*Table 4*).

**Table 4:**
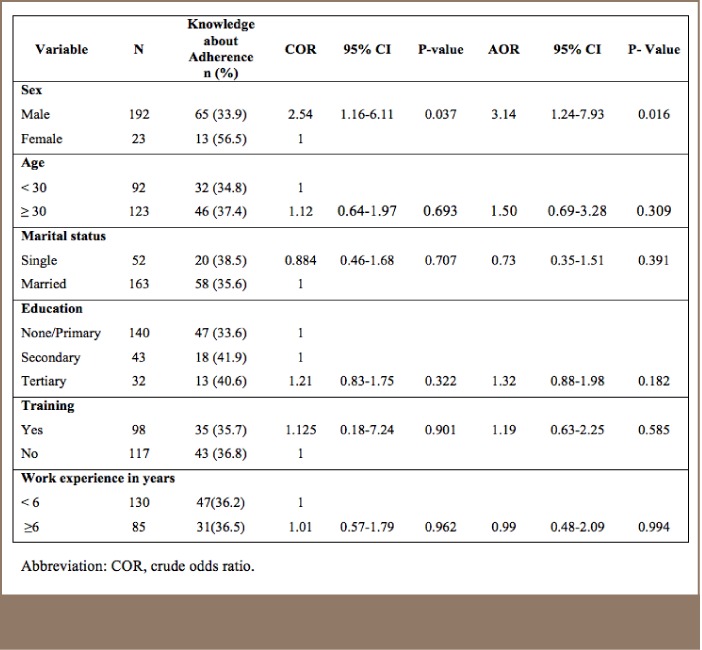
Mine Worker Knowledge About Adherence to the Cyanide Code

### Site Compliance

In terms of site compliance to the Cyanide Code, only 35.3% (n=6) of the SGM sites obtained a score greater than 50%, and hence were classified as adhering to the criteria set for mining for operations using cyanide as a lixiviant (*Supplemental Material 2 and 3*). Among the 19 SGMs enrolled in this study, only 2 (10.5%) obtained a score greater than 80%, indicating a high level of compliance with the Cyanide Code, 4 (21.1%) scored between 50% and 70% suggesting moderate compliance (i.e., failing on some of the checklist and observation items), and 13 (68.4%) sites scored less than 50% and hence were rated as not adhering to the Cyanide Code. Workers' knowledge of Cyanide Code was not associated with the level of site compliance.

Table 5 provides details on cyanide residue and cyanide waste management among the audited SGM sites. Monitoring and control of pH is important for keeping the predominance of cyanide ion (CN-) over hydrogen cyanide (HCN) as the latter can be volatile and is extremely toxic. Only 36.8% (n=7) of the sites had implemented monitoring and control of pH during the leaching process. Furthermore, 47% (n=9) of the SGM sites audited for Cyanide Code compliance were found to be discharging processing wastes into nearby farms and/or rivers. With regard to empty cyanide storage containers, only 21.1% (n=4) of the SGM operations showed evidence that these containers were treated, discarded, and buried underground. The rest of the sites were re-using the containers for different purposes.

**Table 5:**
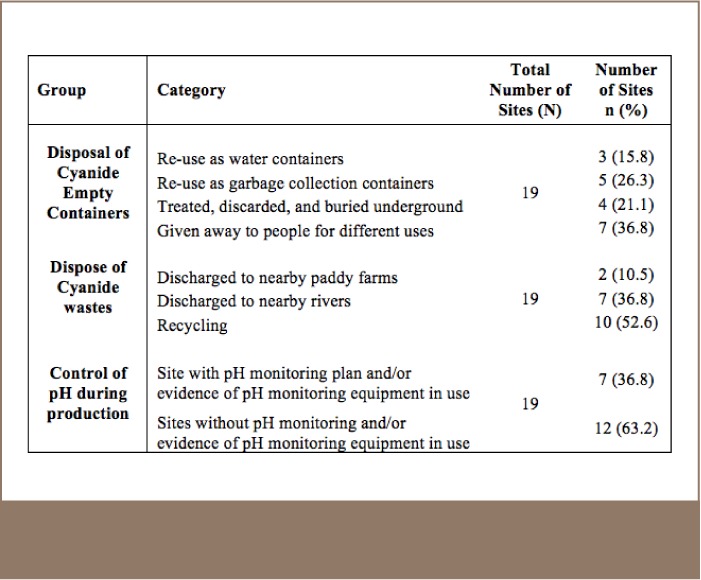
Management of Cyanide Residues and Cyanide Related Wastes Among the Audited Sites

## Discussion

The results of the present study revealed that more than 60% of the mine workers and more than one-third of the mine managers were not knowledgeable about the requirements of the Cyanide Code. Not surprisingly, workers who were not knowledgeable were also less likely to adhere to the Cyanide Code protocols. Consistent with other studies, education and training were found to be the key determinants of an individual's knowledge of the Cyanide Code.^14^ In Zimbabwe, mine workers without cyanide management training were found to be incompetent in handling and using cyanide.^32^ Our findings support the call for strengthening training and compliance to the Cyanide Code in SGM operations in Tanzania and worldwide. This includes the provision of adequate personal protective equipment (PPE), and training in cyanide management and safe methods for handling and disposing of cyanide materials.^14^,^35^

The link between education level and knowledge of the Cyanide Code could be because more educated workers are able to read and understand educational support materials, including safety posters and material safety data sheets posted at the mining sites. However, the low levels of reading ability and education of most workers in SGM operations in Tanzania and worldwide need to be taken into consideration when developing safety posters, data sheets and training programs, and in the labeling that is placed on the cyanide containers. Posters, data sheets, training and labeling should utilize visual representations (i.e., pictures) and associated oral communications should be congruent with these visual representations. Importantly, we observed that all cyanide containers and other associated chemicals at all of the 19 SGM sites in Geita had labels written in English. To improve workers' knowledge, the Tanzanian government should require that all containers and materials come with bilingual labels (English and Swahili) and pictorial representations.

Previous research has suggested that workers' low levels of knowledge and poor adherence to the Cyanide Code could be attributed to reliance on traditional methods in mining activities.^10^,^17^ Also, lack of knowledge about cyanide use and inadequate supervision have been reported as some of the reasons for poor cyanide management in most of the gold mining industries.^14^,^24^ In other studies, lack of monitoring tools and inadequate management skills have been reported to be factors associated with lack of adherence to the Cyanide Code.^14^,^17^ Training of mine workers should be a high priority, as individuals with inadequate training could place themselves and others at significant risk.^16^,^35^ Interestingly, married individuals were found to be more concerned and more knowledgeable of the Cyanide Code compared to unmarried individuals. This could be due to their seniority in the mining industry and/or to their concerns for their own safety, the safety of their families, or the importance of having a secure future livelihood. Even though workers stated that they worked mainly in cyanidation, proccessing or the laboratory, information obtained during the site audit suggested that workers were rotated to different sections of the worksite and performed different jobs depending on where the need was on a specific day. This could be due to the small size of most SGM operations. Individual workers working in the cyanidation unit, the processing plant and the laboratory could be at higher risk of exposure to cyanide compared to support staff. During our assessment of the SGM operations, we did not use human exposure monitors for cyanide. Follow up studies that monitor exposure levels in different sections of SGM mining operations are needed to ensure the safety of workers.

Although almost two-thirds of the mining managers were knowledgeable about the Cyanide Code, this knowledge did not appear to have been translated to the mine workers. This suggests that managers' knowledge of the Cyanide Code did not significantly influence their actions regarding the protection and training of their workers on cyanide exposure. This finding is consistent with those reported by the United Nations Environmental Programme (UNEP), and a study in Zimbabwe that reported discrepancies between managers' and mine workers' knowledge.^5^,^35^ Even when managers reported that workers had been given primary safety gear, such as gloves and safety boots, very few of the mine workers were observed wearing them, and it appears that no actions were taken by the managers to ensure that the workers utilized this gear while on the mine site. Similar findings have been reported in Zimbabwe and Ecuador.^14^,^35^

In the present survey, most SGM sites did not meet the basic Cyanide Code requirements set by the International Cyanide Management Institute. Generally, most of the SGM sites did not have guidelines for cyanide management, and did not provide proper personal protective equipment for their workers. Lack of specifically trained personnel for administering antidotes and other first aid services were other areas in which many SGM operations displayed a lack of compliance to the Cyanide Code. A practice of concern that was observed at many of the SGM sites was the storage of cyanide chemicals together with other chemicals; this could result in increased chemical exposure hazards at these sites. An even greater concern was the lack of emergency procedure guidelines in case of an accident and/or spillage at most of the SGM sites. Similar findings were observed in Brazil where most SGM operations did not comply with Cyanide Code standards resulting in the pollution of rivers and other receiving environments due to improper waste mismanagement at SGM sites.^15^

The massive shift of SGM from rudimentary mercury use to cyanide technology in Tanzania may be seen as an excellent opportunity to minimize the introduction of mercury into the environment. However, there is a significant need to protect mine workers and the surrounding communities from cyanide toxicity and associated cyanide wastes and compliance to the Cyanide Code should therefore be a priority. The reprocessing of rudimentary mercury tailings at SGM sites using cyanide may produce a combined toxic mercury-cyanide complex that results in mercury becoming bioavailable and soluble, and increases the chances for methylation of mercury into its more toxic form.^20^,^21^ Evidence from the literature suggests that for gold processing operations that use cyanidation technology and where there is possibility of mercury contamination, treating wastes with strong precipitants such as sulphides is vital.^21^ Soluble sulphides have the potential to convert all mercury cyanide complexes into insoluble metal sulhides at all pH values, reducing the chance that the mercury methylation process occurs.^20^ However, treatment of wastes with sulphides was not performed at any of the SGM operations in northern Tanzania. Because of the widespread presence of SGM sites in northern Tanzania and their current use of mercury amalgamated tailings, not treating wastes from mines that use cyanide technology could result in the spread of mercury beyond the original mining sites and into communities that are directly involved in mining activities.

The introduction of cyanidation technology into SGM was in response to its increased efficiency in recovering gold compared to amalgamation.^36^,^37^ This has resulted in the industry changing from using one toxic chemical—mercury—to another—cyanide—for processing and recovering gold.^36^,^37^ Efforts within the industry have emphasized compliance with the Cyanide Code; however, there is a need to develop and implement alternative safe mining solutions. Studies from the Phillipines and Zimbabwe have reported on the successiful introduction and operation of a mercury and cyanide free technique—the gravity-borax method.^36^,^37^ The gravity-borax method uses the same equipment as the amalgamation technique, is more efficient with higher gold recovery compared to cyanidation, and much safer to the environment and human health.^36^,^37^

The findings of this study strongly suggest that stakeholders in SGM in Tanzania should strengthen compliance to occupational health, environmental and chemical standards as articulated in the Cyanide Code, as well as explore opportunities beyond pure regulatory approaches for improving the situation, including the introduction of the gravity-borax method. There is also a need to engage the broader artisanal mining communities as SGM operations, the artisanal miner, and Tanzania agencies and authorities share responsibility for environmental stewardship and the protection of human health. For example, the Tanzania Mining Agency National Environmental Management Council should take a more active role in ensuring adherence to the Cyanide Code. In addition, the findings of the present study indicate that greater emphasis must be placed on ensuring that mine workers and managers recieve training on and comply with the Cyanide Code.

This is the first study in Tanzania to examine knowledge of and adherence to the Cyanide Code among SGM workers and managers. Although the findings cannot be generalized to other parts of the country or worldwide, they support the contention that there is a need to develop capacity for training programs on worker safety, environmental protection, and community health. Furthermore, while regulatory enforcement is important, a multifaceted approach to improving worker safety and environmental health is needed, including the development of innovative interventions encouraging the participation and involvement of miners. We also recommend in-depth group discussions among mine workers and managers in order to better to understand their lived experience of occupational and environmental health issues related to cyanide exposure in SGM sites. Finally, it is important to note that even though all the SGM mining sites were physically visited during the study, some potentially hazardous mining practices may have not been revealed and/or observed as managers and mine workers might have been concerned about possible site closure and/or being penalized by regulatory authorities in case the information collected was disclosed. This should be considered as one of the key limitations of the present study.

## Conclusions

The present study found that SGM workers in Tanzania have limited knowledge of and report poor adherence to the Cyanide Code. There is a need for further research on government participation in and enforcement of the Cyanide Code in Tanzania, especially in SGM operations. To minimize potential cyanide toxicity among the workers in the local communities and the general environment, clear strategies that are agreed upon and supported by stakeholders at all levels are required.
