# Supplementary material for: Knowledge of and Adherence to the Cyanide Code Among Small-scale Gold Miners in Northern Tanzania
Source: J Health Pollut. 2017 Jun 22;7(14):4–14. doi: 10.5696/2156-9614-7.14.4 (PMC6259476; doi:10.5696/2156-9614-7.14.4)
Supplement: Supplementary file 1 [file NyanzaSupplemental.docx]

# Supplemental Material 1:

# Cyanide Code knowledge questionnaire

| **S/N** | **Question** | **Categories** | **Score** |
| --- | --- | --- | --- |
|  | What is the Cyanide Code? | Guidelines used by cyanide users to manage cyanide at their workplaces. | 2 |
|  |  | Guideline leaflets with some instructions on safe means of handling cyanide. |  |
|  |  | Book prepared especially for cyanide users on how to use cyanide. |  |
|  |  | I don’t know. | 0 |
|  | What are the benefits of using the code in the management of cyanide spillage in your work section? | It provides steps to follow on how to deal/handle cyanide spillage without endangering people’s health or that of other species. | 2 |
|  |  | It is a tool which helps cyanide users make decisions regarding cleaning of cyanide spillage. |  |
|  |  | It provides standards which need to be attained after cleaning the spillage. | 0 |
|  | In case of cyanide poisoning what are the procedures to help people who: | Use of antidote as instructed by doctor. | 2 |
|  | i) Ingested cyanide | Use of medication from first aid kit. |  |
|  | ii) Skin contacted with cyanide | Wash with water for 15 minutes. |  |
|  | iii) Inhaled cyanide | Wash with water. |  |
|  | iv) Eye contacted cyanide | Place affected person to the area which is well ventilated. |  |
|  |  | I don’t know. | 0 |
|  | Please describe personal protective gear for protecting yourself from cyanide exposure. | Plastic aprons, gas masks, PVC gloves, safety boots. | 2 |
|  |  | Reflectors, uniforms, gumboots. | 0 |
|  | What are the procedures for disposing of cyanide-contaminated materials? | Decontamination prior to disposal. | 2 |
|  |  | Seeking permission from Env. Dept prior to disposal of contaminated materials. |  |
|  |  | I don’t know. | 0 |
|  | Please explain cyanide storage conditions. | Store in ventilated rooms, avoid storing cyanide in direct sunlight and store cyanide in separate room with other chemicals, usage of PPE during storage of cyanide. | 2 |
|  |  |  |  |
|  |  | I don’t know. | 0 |
|  | What potential harm can you cause due to failure to follow cyanide code procedures? | Poisoning of people using cyanide which may lead to diseases like skin pigmentation, possible death of people and other living organisms, environmental pollution. | 2 |
|  |  | I don’t know. | 0 |

Supplemental Material 2:

Knowledge of Cyanide Code adherence

| **S/N** | **Questions on adherence to Cyanide Code** |
| --- | --- |
|  | How do mining workers protect themselves from cyanide exposure in the workplace? |
|  | How are the cyanide empty containers managed? |
|  | How does cyanide residual get destroyed after completion of the extraction process? |
|  | How is pH controlled during gold extraction (for managers)? |
|  | How is cyanide airborne gas monitored? |
|  | Do you use appropriate protective gear in the work section (list the gear in use)? |
|  | How often is protective gear in use? |
|  | Is an antidote used in case of cyanide ingestion or inhalation? |
|  | Are you trained to use the antidote - a substance which counteract the harmful effects of cyanide? Provide proof of attendance at a training. |
|  | What preventive measures exist to protect the people and the community around the mine? |

Supplemental Material 3:

Site audit checklist for examining adherence and compliance to the Cyanide Code adapted with modification of the **International Cyanide Management Code**

| **S/N** | **Question** |
| --- | --- |
| 1 | Is there a written cyanide management strategy in place? |
| 2 | Are training guidelines posted? |
| 3 | Are risks and responses posted for workers for cyanide accidents? |
| 4 | Are there procedures for safe storage of cyanide posted? |
| 5 | Has a risk assessment of the workplace been undertaken? Is this available? |
| 6 | Are there procedures in place for clean-up in the event of accidental spillage? Is there written signage and documentation? |
| 7 | If they exist, do clean-up procedures specify standards to be achieved for environmental protection, including environmentally responsible disposal of contaminated materials? |
| 8 | Does the mine closure plan address cyanide disposal include disposal of contaminated materials? |
| 9 | Does the mine closure plan include specifications of and provisions for long term monitoring of cyanide levels? |
| 10 | Is there a monitoring regime in place which addresses all of the possible areas and pathways of environmental impact from cyanide and its various forms? |
| 11 | Are worker health and environmental protection targets set appropriate to the different forms of cyanide? |
| 12 | Does the monitoring regime specify sampling protocols appropriate to the different forms of cyanide? |
| 13 | Does the monitoring regime specify appropriate layout, techniques, frequency, quality and sensitivity of monitoring and sampling? |
| 14 | Are data collected in accordance with the requirements of the monitoring regime? |
| 15 | Are the data analysed and regularly reported to the regulatory authorities? |
| 16 | Are noncompliance issues or abnormalities in the data routinely investigated? |
| 17 | Are emergency procedures in place with specific measures for managing cyanide? |

**Supplemental Material 4:
Site observation for SGM regarding adherence to the Cyanide Code, adopted from
Rosia Montana Gold Corporation.^34^**

| S/N | Observation |
| --- | --- |
|  | Are all cyanide tanks, containers and piping clearly labeled as containing cyanide? |
|  | Is the direction of flow indicated on all cyanide piping? |
|  | Are there signs of corrosion or deterioration on any cyanide tanks or containers? |
|  | Is personal protective equipment available and in good condition? |
|  | Are HCN monitor/alarms in working conditions? |
|  | Are fire extinguishers charged and in good working order? |
|  | Is there any evidence of leakage or spillage from cyanide tanks and containers? |
|  | Is any water or solution present in cyanide secondary containment? |
|  | Are cyanide secondary containment walls and flow separated, cracked or deteriorated? |
|  | Are emergency alarms in good working condition? |
|  | Is there spill response or communication equipment ready available? |

**Supplemental Material 5:**
**Operations at an SGM site in northern Tanzania**(*Photo Cortesy of E. Nyanza & D. Dewey)*

| 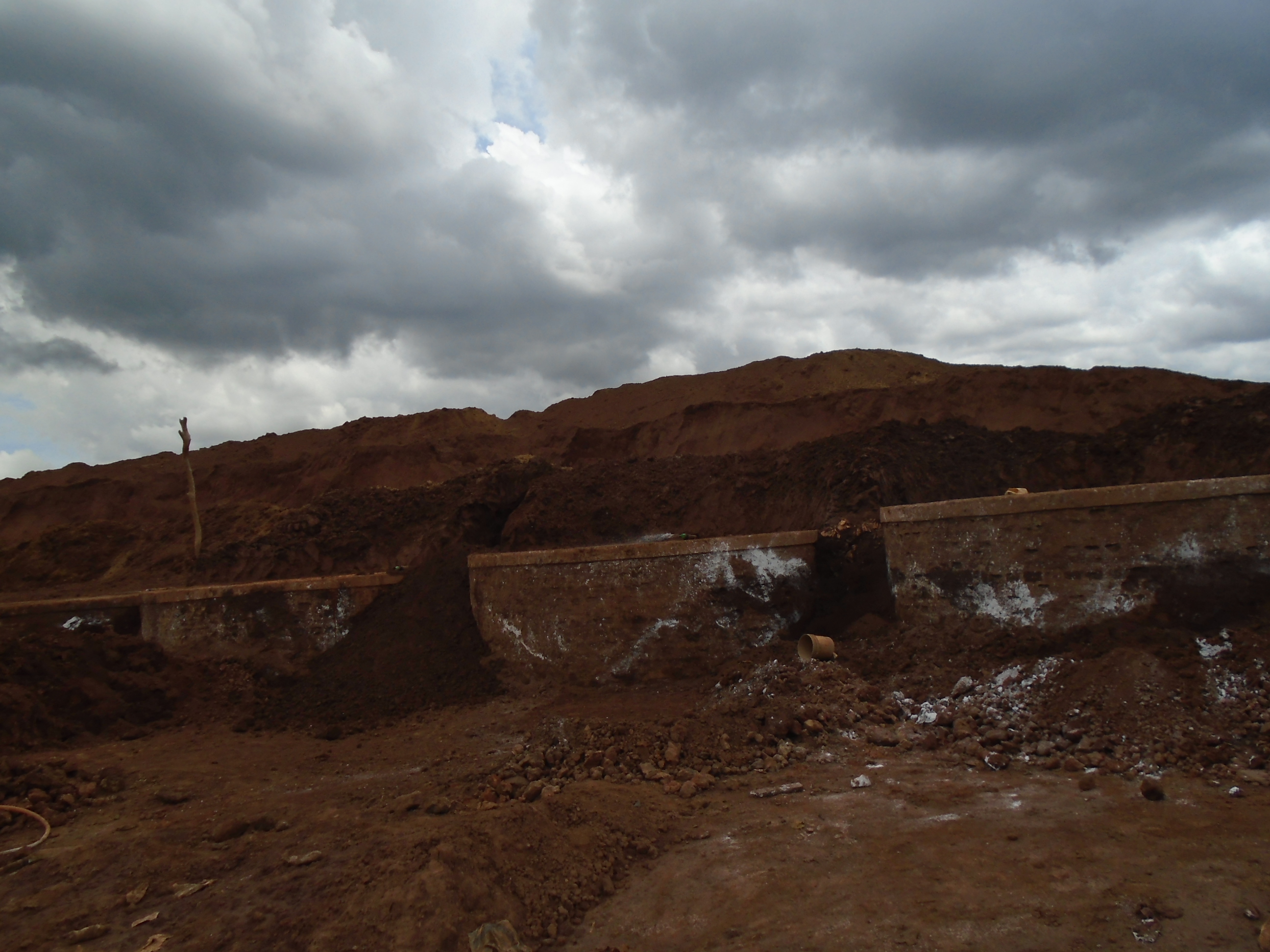  (a). Cyanide leaching tanks, with tailings piled up after processing. | | 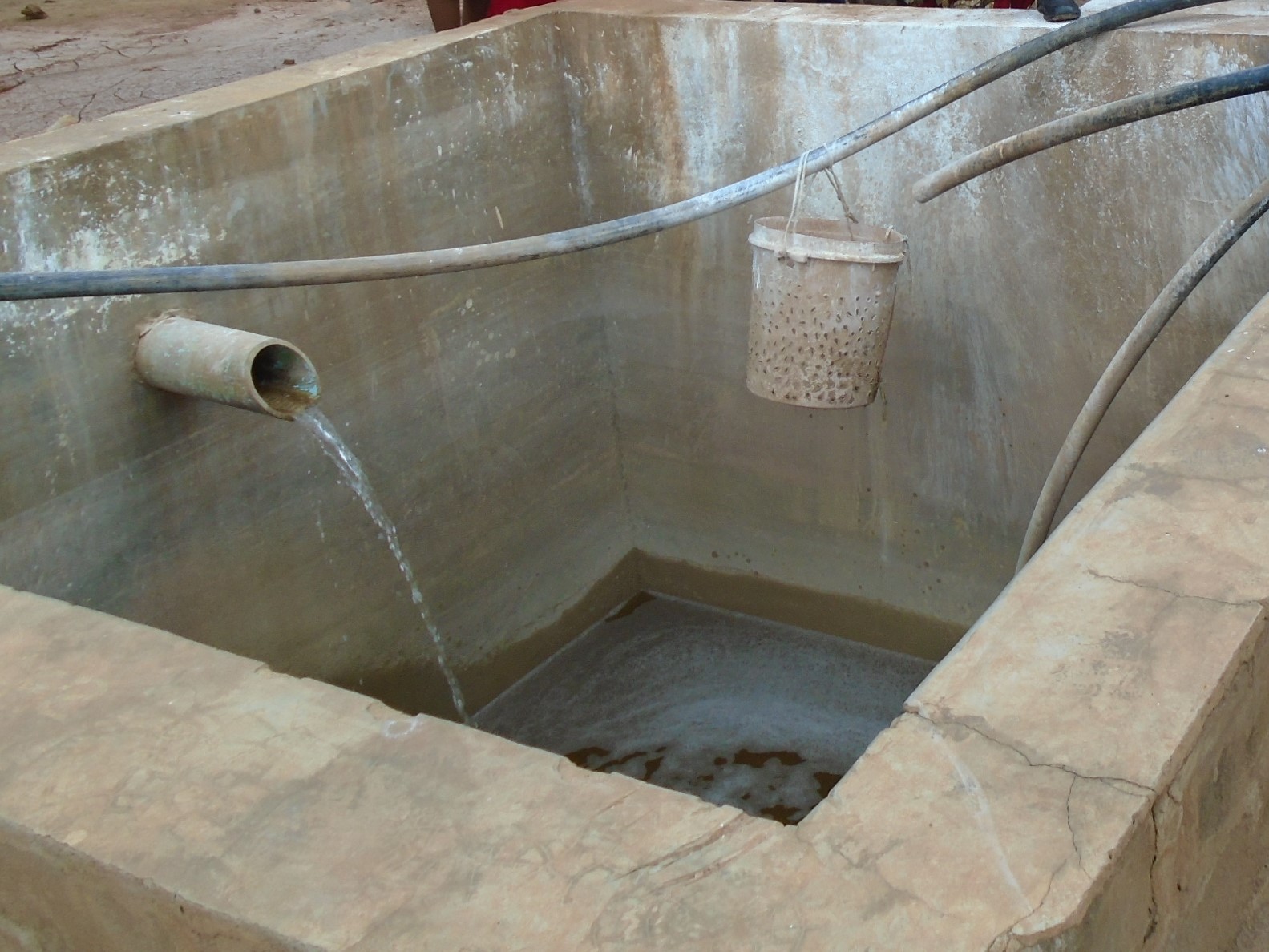  (b). Preparation of cyanide solution in an open tank to make 1000 ppm concentration. | |
| --- | --- | --- | --- |
| 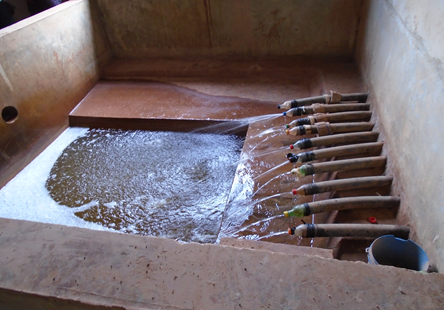  (c). Leaching chamber where first gold absorbtion using carbon materials happens. A series of similar chambers follows as in (d); each drainage pipe represents one leaching tank. | 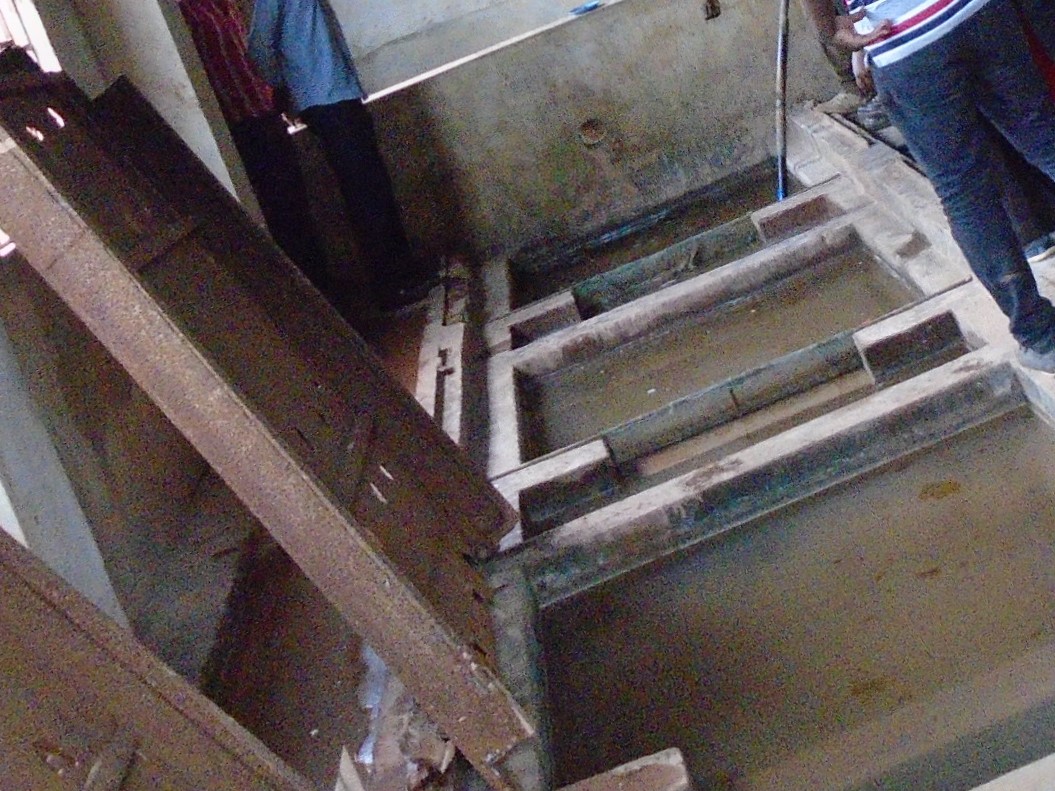  (d). A series of chambers with carbon materials to absorb gold tracked in cyanide complexes. | | 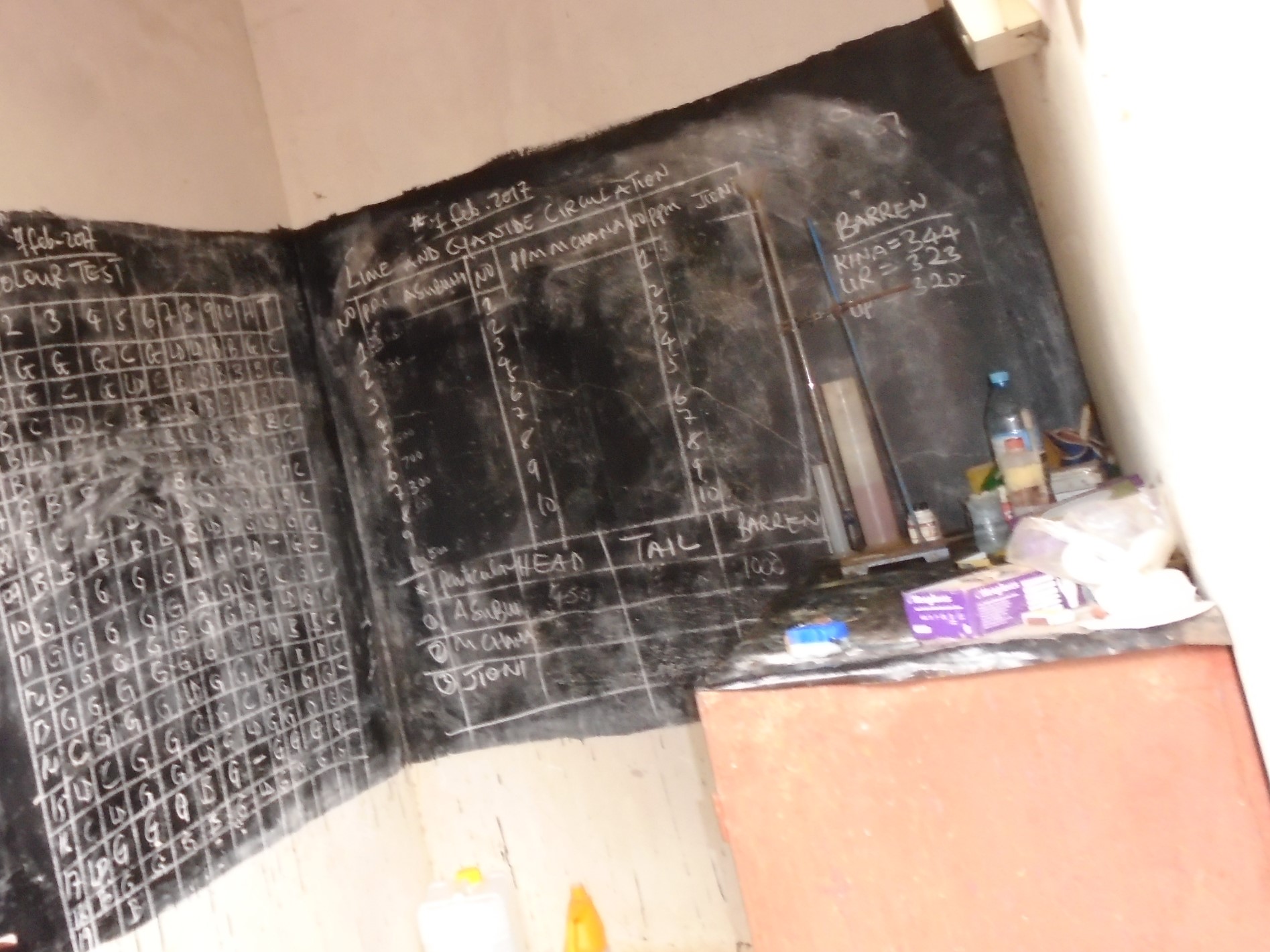  (e). Laboratory site for checking the concentration of gold remaining in the leaching tanks (d), and for ensuring 1000 ppm of cyanide solution is made in (b) above. |
